# A combined genetic and bioinformatic analysis implicates the *CDKN1A* rs1059234 variant in differentiated thyroid carcinoma susceptibility

**DOI:** 10.20945/2359-4292-2026-0119

**Published:** 2026-10-11

**Authors:** Elisangela de Souza Teixeira, Raquel Bueno Barbieri, Larissa Teodoro Rabi, Karina Colombera Peres, Janaina Luisa Leite, Natassia Elena Bufalo, Laura Sterian Ward

**Affiliations:** 1 Laboratório de Genética Molecular do Câncer, Faculdade de Ciências Médicas, Universidade Estadual de Campinas, Campinas, SP, Brasil; 2 Faculdade de Medicina, Centro Universitário Max Planck, Indaiatuba, SP, Brasil; 3 Departamento de Patologia, Instituto e Centro de Pesquisas São Leopoldo Mandic, Campinas, SP, Brasil

**Keywords:** *CDKN1A*, *CDKN1B*, genetic polymorphism, differentiated thyroid carcinoma, cell cycle regulation, susceptibility variant

## Abstract

**Objective:**

To evaluate the association of *CDKN1A* (rs1801270 and rs1059234) and *CDKN1B* (rs2066827 and rs34330) polymorphisms with differentiated thyroid carcinoma (DTC) risk and to explore potential functional impacts through *in silico* analyses.

**Subjects and methods:**

This case-control study included 185 DTC patients and 236 healthy controls. Genotyping was performed using TaqMan® assays, and associations with DTC risk and clinicopathological features were assessed via logistic regression. Bioinformatic tools were used to predict the structural and regulatory effects of the studied polymorphisms.

**Results:**

The *CDKN1A* rs1059234 polymorphism showed a statistically significant association with increased DTC risk, particularly for the CT genotype (odds ratio = 6.575; 95% confidence interval: 4.004-10.803; *p* < 0.0001). *In silico* analyses suggested that rs1059234 may modify microRNA binding sites within the 3'UTR, potentially modifying the post-transcriptional regulation of *CDKN1A.* This variant was not correlated with tumor characteristics, including tumor size, histological subtype, or meta-static status. No significant associations were found for rs1801270, rs2066827, or rs34330. Predictions for missense variants (rs1801270 and rs2066827) were inconsistent, with some tools indicating possible destabilizing effects on p21 or p27 protein structure.

**Conclusion:**

The *CDKN1A* rs1059234 polymorphism may represent a candidate susceptibility marker for DTC, possibly through altered post-transcriptional regulation of p21. However, due to the moderate sample size and the absence of functional validation, these findings should be considered hypothesis-generating and require independent replication in larger cohorts with experimental validation. The remaining investigated variants did not meaningfully influence DTC risk in this study.

## INTRODUCTION

The global incidence of thyroid cancer has risen substantially over recent decades, underscoring the need to better understand its molecular determinants and to identify biomarkers for improved risk assessment and early detection (^1^,^2^). Although most cases are sporadic, approximately 5-15% of non-medullary thyroid carcinomas exhibit familial aggregation, suggesting a heritable component (^3^,^4^). Genetic predisposition to non-medullary thyroid carcinomas reflects a complex architecture involving both rare high-penetrance mutations and common low-penetrance variants (^5^). Despite advances from genome-wide association studies and targeted sequencing, much of the inherited risk remains unexplained (^6^), emphasizing the importance of investigating additional candidate variants.

Disruption of cell cycle control is a hallmark of carcinogenesis (^7^). Among key regulators of G1-phase progression are cyclin-dependent kinases (CDKs) and their inhibitors, particularly p21 (encoded by *CDKN1A)* and p27 (encoded by *CDKN1B),* which act as **criti**cal checkpoints responsible for maintaining genomic stability (^8^). Impairment of p21 or p27 function may facilitate uncontrolled proliferation and favor malignant transformation. The *CDKN1A* gene encodes p21, a central mediator of p53-dependent growth arrest and DNA damage response (^9^,^10^). Functional or regulatory disruption of this protein has been implicated in various malignancies. Two commonly studied *CDKN1A* variants are the missense polymorphism rs1801270 (Ser31Arg), previously associated with a reduced risk of medullary thyroid carcinoma (^11^), and the 3'UTR variant rs1059234, which may affect mRNA stability (^12^) and has been associated with susceptibility in gastric and esophageal (^13^,^14^,^15^,^16^). Nonetheless, their involvement in thyroid tumorigenesis remains unclear.

Similarly, *CDKN1B* encodes p27, another important CDK inhibitor whose reduced expression or abnormal subcellular localization has been linked to aggressive tumor behavior in thyroid cancer (^17^,^18^,^19^). Genetic variability in *CDKN1B* may modulate susceptibility or gene expression, including the promoter polymorphism rs34330, which alters transcript levels (^20^), and germline mutations associated with endocrine tumor syndromes such as MEN4 (^21^).

Given the central roles of p21 and p27 in cell cycle regulation, variants in *CDKN1A* and *CDKN1B* are biologically plausible contributors to thyroid cancer predisposition. However, existing data are limited and inconsistent. Therefore, this study sought to investigate the association of *CDKN1A* (rs1801270 and rs1059234) and *CDKN1B* (rs2066827 and rs34330) polymorphisms with differentiated thyroid carcinoma (DTC) risk, integrating case-control genotyping with *in silico* interpretations of these analyses to elucidate possible mechanisms. Identifying genetic variants that modulate DTC risk could ultimately improve risk assessment and inform clinical strategies, including individualized surveillance and early intervention in high-risk individuals.

## SUBJECTS AND METHODS

### Study population

This case-control study included 421 participants and was conducted in accordance with the Declaration of Helsinki. Written informed consent was obtained from all individuals, and the protocol was approved by the Research Ethics Committee of the School of Medical Sciences of the University of Campinas (approval no. 1088/2011). The study population comprised 185 patients with DTC and 236 healthy controls. Among the patients, 141 were women and 44 were men, with a mean age of 42.1 ± 10.4 years. Histological examination identified 95 cases of papillary thyroid carcinoma and 19 cases of follicular thyroid carcinoma. Comprehensive clinical, surgical, and histopathological data, including tumor size, histologic subtype, and lymph node status, were available for all patients. Control subjects (173 women and 63 men, mean age 39.5 ± 12.6 years) were recruited from volunteer blood donors at the Hematology and Hemotherapy Center of the University of Campinas. None had personal or family histories of cancer or thyroid disease, benign thyroid nodules, or autoimmune thyroiditis.

### DNA extraction and genotyping

Peripheral blood samples were collected in EDTA tubes, and genomic DNA was extracted using standard phenol-chloroform protocols. DNA concentration and purity were assessed by ultraviolet spec-trophotometry (Picodrop, Cambridgeshire, UK), and samples were stored at -20 °C until analysis. Genotyping used TaqMan^®^ SNP Genotyping Assays (Applied Biosystems) on a 7500 Real-Time PCR System (Applied Biosystems, USA). The four selected single nucleotide polymorphisms (SNPs; *CDKN1A* rs1801270 and rs1059234; *CDKN1B* rs2066827 and rs34330) were chosen based on prior evidence of cancer associations and were confirmed to lack strong linkage disequilibrium, as assessed with HaploView and HapMap data. Polymerase chain reaction was performed in a 7 µL reaction containing 20 ng genomic DNA, 1× TaqMan® Genotyping Master Mix, 40×SNP assay (probes and primers), and nuclease-free water. Thermal cycling conditions were: 95 °C for 10 min, followed by 50 cycles of 92 °C for 15 s and 60 °C for 90 s. Genotypes were automatically assigned using TaqMan® Genotyper Software (v. 1.3) and classified as wild-type homozygous, heterozygous, or variant homozygous. Call rates exceeded 95% for all four SNPs. Negative controls (no template) were included in each run; 5% of samples were randomly selected for duplicate genotyping, yielding 100% concordance.

## BIOINFORMATICS ANALYSIS

### Missense variants

To evaluate the functional consequences of the missense SNPs rs1801270 *(CDKN1A)* and rs2066827 *(CDKN1B),* we obtained the protein sequences from UniProt (p21: P38936; p27: P46527). *In silico* predictions included SIFT, BLINK, and PolyPhen-2 for tolerance to amino acid substitution; SNPs&GO, PhD-SNP, and PMut for pathogenicity prediction; and I-Mutant 2.0 and MUpro for protein stability assessment (^22^,^23^,^24^,^25^,^26^).

### UTR variants

For the 3'UTR (rs1059234) and 5'UTR (rs34330) variants, we obtained functional annotations using the HaploReg tool (v. 4.2) for regulatory elements and chromatin states, as well as rSNPBase for evaluating transcriptional and post-transcriptional regulatory potential (^27^,^28^). PolymiRTS was used to assess alterations in microRNA binding motifs (^29^).

### Evolutionary conservation and structural modeling

The evolutionary conservation of amino acid residues was analyzed using ConSurf, which assigns conservation scores based on comparative genomics. Structural stability predictions for missense variants were generated using DynaMut2, providing ΔΔG values and structural modeling to infer potential impacts on protein folding and dynamics (^30^,^31^).

### Statistical analysis

Statistical analyses were performed using SAS software (v. 9.4; SAS Institute, USA). Continuous variables were expressed as mean ± standard deviation and compared using the Mann-Whitney or Kruskal-Wallis tests, as appropriate. Categorical variables were analyzed with the Chi-square test or Fisher's exact test.

Hardy-Weinberg equilibrium was assessed for all SNPs in the control group. Associations between genotypes and DTC risk were estimated using odds ratios (ORs) with 95% confidence intervals (CIs). Both univariate and multivariate logistic regression analyses were performed using backward stepwise selection to identify independent predictors. Given the exploratory nature of this study and the presence of four *a priori* hypotheses, no formal correction for multiple comparisons was applied in order to avoid type II error. Therefore, these findings should be regarded as hypothesis-generating and require independent replication in future studies.

## RESULTS

All SNPs were in Hardy-Weinberg equilibrium in the control group (rs1801270: *p* = 0.21; rs1059234: *p* = 0.34; rs2066827: *p* = 0.18; rs34330: *p* = 0.45). Genotyping success rates were 385/421 participants (91.4%) for rs1059234, 365/421 (86.7%) for rs1801270, 408/421 (96.9%) for rs2066827, and 417/421 (99.0%) for rs34330. **Table 1** summarizes the genotype distribution for the four investigated variants (rs1801270 and rs1059234 in *CDKN1A;* rs2066827 and rs34330 in *CDKN1B),* along with the participants' demographic and clinicopathological characteristics. Patients and controls differed slightly in age (42.1 vs. 39.5 years, *p* = 0.0449), which was accounted for in subsequent analyses. Sex distribution and other demographic variables did not differ significantly between groups. **Figure 1** shows the overall genotype distributions for each SNP. Some samples did not yield clear genotyping results; therefore, the final sample sizes varied slightly between variants.

**Table 1:** Genotypic distributions of the rs1801270, rs1059234, rs2066827, and rs34330 polymorphic variants compared to the homozygous major allele according to the clinical and pathological characteristics of the 184 thyroid nodules analyzed

**Clinical and anatomopathological characteristics**		**rs1801270**		***P***	**rs1059234**		***P***	**rs2066827**		***P***	**rs34330**		***P***
		**AA**	**AC+CC**		**TT**	**CT+CC**		**GG**	**TG+CC**		**CC**	**CT+TT**	
Age (years), mean ± SD		42.0±10.7	42.9±11.0	0.935	42.5±10.8	43.1±10.9	0.563	40.4±12.2	42.1±10.6	0.718	40.9±11.5	40.4±12.0	0.312
Sex	Female	103 (76.9)	31 (23.1)	0.879	46 (32.6)	95 (67.4)	0.981	73 (51.8)	68 (48.2)	0.191	76 (54.3)	64 (45.7)	0.1376
	Male	28 (75.7)	9 (24.3)		14 (31.8)	30 (68.2)		25 (56.8)	19 (43.2)		29 (65.9)	15 (34.1)	
Tumor size (cm), mean ± SD		2.39±1.64	3.01±1.99	0.183	2.1±1.4	2.7±1.9	0.346	2.3±1.6	2.7±1.8	0.163	2.4±1.6	2.6±1.9	0.214
Variant	Papillary	95 (77.9)	27 (22.1)	0.405	26 (19.7)	106 (80.3)	0.176	68 (51.5)	64 (48.8)	0.498	6 (4.5)	126 (95.4)	0.241
	Follicular	19 (70.9)	8 (29.6)		9 (33.3)	18 (66.6)		16 (59.3)	11 (40.7)		3 (11.1)	24 (88.9)	
Capsular invasion	Absent	54 (76.9)	17 (23.9)	0.273	29 (37.7)	48 (62.7)	0.295	41 (53.2)	36 (46.8)	0.809	1 (1.3)	76 (98.7)	0.080
	Present	24 (64.9)	13 (86.7)		12 (30.8)	27 (69.2)		17 (43.6)	22 (56.4)		3 (7.7)	36 (92.3)	
Lymphatic invasion	Absent	54 (76.9)	17 (23.9)	0.260	29 (37.7)	48 (62.7)	0.29	41 (53.2)	36 (46.8)	0.809	1 (1.3)	76 (98.7)	0.080
	Present	13 (86.7)	2 (13.3)		4 (25.0)	12 (75.0)		10 (62.5)	6 (37.5)		3 (18.8)	13 (81.3)	
Multifocality	Absent	68 (76.4)	21 (23.6)	0.993	33 (34.7)	62 (65.3)	0.773	49 (51.6)	46 (48.4)	0.428	6 (6.3)	89 (63.7)	0.511
	Present	39 (76.5)	12 (23.5)		17 (32.0)	36 (68.0)		25 (47.2)	28 (52.8)		2 (3.8)	51 (96.2)	
Metastasis at diagnosis	Absent	68 (76.4)	21 (23.6)	0.868	21 (22.1)	74 (77.9)	0.943	50 (52.6)	45 (47.4)	0.801	6 (6.3)	89 (93.6)	0.910
	Present	45 (77.6)	13 (22.4)		13 (21.3)	48 (78.7)		32 (52.5)	29 (47.6)		3 (4.9)	58 (95.1)	

Data are presented as n (%) unless otherwise noted.

**Figure 1:**
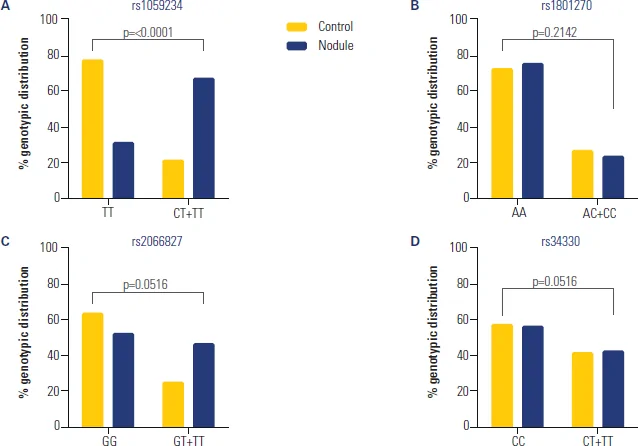
Genotypic distributions of the four single nucleotide polymorphisms in differentiated thyroid carcinoma patients and controls (Fisher's exact test). **(A)** rs1059234; **(B)** rs1801270; **(C)** rs2066827; **(D)** rs34330. Statistically significant differences between groups were observed only for rs1059234 (*p* < 0.0001).

### Genetic association findings *CDKN1A* rs1059234

Genotype frequencies of rs1059234 differed significantly between DTC patients and controls (*p* < 0.0001) **(Figure 1A).** The CT genotype was markedly more frequent among patients, demonstrating a statistically significant association with DTC risk (OR = 6.576; 95% CI: 4.004-10.803; *p* < 0.0001). The TT genotype was also associated with increased risk (OR = 10.140; 95% CI: 4.762-21.592; *p* < 0.0001). The main ORs for rs1059234 are summarized in **Table 2,** with the genotype-specific comparisons further detailed in **Table 3.** No significant associations were found between rs1059234 genotypes and tumor size, histological subtype, capsular or lymphatic invasion, multifocality, or metastatic presentation at diagnosis (all *p* > 0.05) **(Table 1).**

**Table 2:** Logistic Regression Analysis of Differentiated Thyroid Carcinoma Risk Associated with *CDKN1A* and *CDKN1B* Polymorphisms, Demographic, and Lifestyle Factors

**Variable**	**n**	**Crude OR (95% CI)**	***P***	**Adjusted OR (95% CI)**	P*
Age (continuous)	421	1.020(1.003-1.037)	**0.0223**	1.019(1.001-1.037)	**0.0381**
Sex (female vs. male)	421	1.167(0.748-1.820)	0.4961	1.141(0.718-1.814)	0.5752
Smoking (yes vs. no)	421	4.900(2.752-8.725)	**<0.0001**	4.872(2.701-8.789)	**<0.0001**
rs1801270 (Ref.: AA)	365		0.2716		0.2983
AC		5.104(0.590-14.175)		4.987(0.571-43.562)	
CC		5.611(0.667-17.217)		5.502(0.649-46.623)	
rs1059234(Ref.: CC)	385		**<0.0001**		**<0.0001**
CT		6.576(4.004-10.803)		6.512(3.952-10.732)	
TT		10.140(4.762-21.592)		10.018 (4.691-21.398)	
rs2066827 (Ref.: GG)	408		0.0528		0.0612
TG		1.373(0.660-2.856)		1.351(0.641-2.847)	
TT		0.806(0.402-1.617)		0.798(0.393-1.621)	
rs34330 (Ref.: TT)	417		0.9950		0.9912
CC		0.997(0.435-2.287)		0.989(0.427-2.291)	
CT		1.018(0.434-2.386)		1.011(0.426-2.401)	

CI: Confidence interval; OR: odds ratio. *Adjusted for age, sex, and smoking status.

**Table 3:** Summary of the association between *CDKN1A* rs1059234 and DTC risk

**Genotype comparison**	**OR (95% CI)**	***P***
CT vs. CC	6.576 (4.004-10.803)	<0.0001
TT vs. CC	10.140 (4.762-21.592)	<0.0001
Dominant model (CT+TT vs. CC)	7.214 (4.516-11.523)	<0.0001

CI: Confidence interval; OR: odds ratio.

### *CDKN1A* rs1801270

Genotype frequencies for rs1801270 did not differ significantly between cases and controls (*p* = 0.2142) **(Figure 1B).** Likewise, there were no significant associations with tumor size, histological type, capsular invasion, lymphatic invasion, multifocality, or metastasis at diagnosis **(Table 1).**

### *CDKN1B* rs2066827

Although the difference in rs2066827 genotype frequencies between patients and controls approached significance (*p* = 0.0516; **Figure 1C),** logistic regression analysis did not indicate a statistically significant association with DTC **(Table 2).** Within the patient group, rs2066827 genotypes were not associated with age, sex, tumor size, capsular invasion, lymphatic invasion, multifocality, or metastasis at diagnosis.

### *CDKN1B* rs34330

Genotype frequencies for rs34330 (CC, CT, and TT) were similar between patients and controls *(p* = 0.2142; **Figure 1D),** and logistic regression confirmed the absence of an association with DTC risk **(Table 2).** No associations were found between rs34330 genotypes and clinical or pathological features, including histological subtype.

### Univariate logistic regression analysis

**Table 2** presents the results of the univariate analysis. Increasing age was associated with a modest but significant increase in DTC risk (OR = 1.020 per year; 95% CI: 1.003-1.037; *p* = 0.0223). Smoking status was also strongly associated with DTC (OR = 4.900; 95% CI: 2.752-8.725; *p* < 0.0001). Of the genetic variants analyzed, only rs1059234 showed a significant association with DTC risk. No significant effects were observed for rs1801270, rs2066827, or rs34330.

### *In silico* functional analysis *CDKN1A* variants *rs1801270 (S31R)*

Bioinformatic predictions for this missense variant were inconsistent. SIFT, PolyPhen-2, SNPs&GO, and PMut classified the substitution as benign or neutral **(Table 4).** Conversely, MUpro and DynaMut2 indicated a destabilizing effect on protein structure (ΔΔG = -0.549 and -0.1 kcal/mol, respectively), whereas I-Mutant 2.0 predicted increased stability. These discrepancies likely reflect differences in algorithm design: SIFT relies on sequence conservation, PolyPhen-2 incorporates structural data, and stability predictors use distinct thermodynamic models, leading to variable outcomes. Conservation analysis with ConSurf gave a score of 6, indicating that the substitution occurs in a region of intermediate to high functional relevance **(Figure 2).**

**Table 4:** Functional analysis results of the *CDKN1A* and *CDKN1B* genes

**ID (AA)**	**SIFT Blink**	**SIFT score**	**PolyPhen2**	**PolyPhen score**	**SNP&GO**	**RI**	**PhD-SNP**	**RI**	**PMut**
rs1801270(S31R)	N	1	B	0	B	10	N	9	B
rs1059234	-	-	-	-	-	-	-	-	-
rs2066827(V109G)	D	0	B	0	B	9	N	7	B
rs34330	-	-	-	-	-	-	-	-	-

A: Amino acid alteration; N: neutral; B: benign; D: deleterious; RI: reliability index.

**Figure 2:**
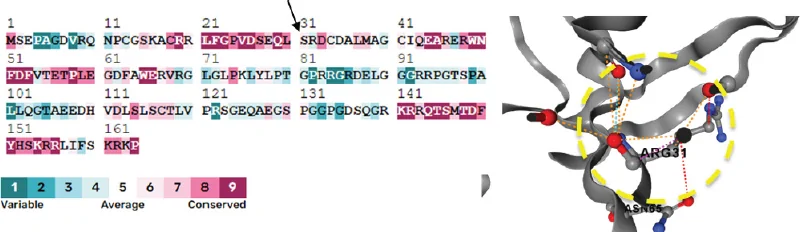
Conservation analysis of *CDKN1A* (p21) generated using ConSurf. Conservation scores range from 1 (variable, light blue) to 9 (highly conserved, magenta). The serine (S) to arginine (R) substitution at position 31 (indicated by the arrow) occurs in a region with a conservation score of 6, indicating intermediate to high conservation. This finding suggests that changes at this position may affect protein function.

### rs1059234 (3'UTR)

PolymiRTS analysis predicted that the ancestral C al-lele binds eight miRNAs, whereas the T allele disrupts these interactions and creates three new miRNA binding sites, suggesting allele-specific modulation of *CDKN1A* post-transcriptional regulation.

### *CDKN1B* variants rs2066827 (V109G)

Functional predictions were again inconsistent. SIFT classified the substitution as deleterious, whereas PolyPhen-2, SNPs&GO, PMut, and PhD-SNP predicted a benign or neutral effect **(Table 4).** MUpro and I-Mutant 2.0 both predicted reduced protein stability (ΔΔG = -2.459). ConSurf indicated that V109G occurs in a non-conserved region of p27 **(Figure 3).**

**Figure 3:**
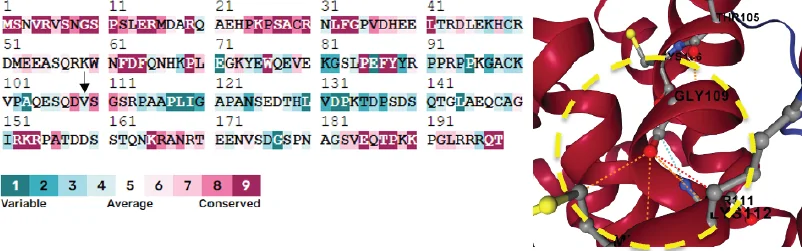
Conservation analysis of *CDKN1B* (p27) using ConSurf. Conservation scores range from 1 (highly variable, light blue) to 9 (highly conserved, magenta). The arrow indicates the V109G (valine-to-glycine) substitution, located in a region of low conservation (scores 1-2), indicating that this position is tolerant to variation.

### rs34330 (5'UTR)

HaploReg annotations showed chromatin signatures consistent with promoter activity (H3K4me3 and H3K27ac). The rSNPBase classified rs34330 as a regulatory SNP with potential proximal effects on *CDKN1B* transcription and possible distal regulatory interactions via RNA-binding proteins. As this is a UTR variant, no effect on amino acid conservation was expected.

### Protein-protein interaction network

STRING network analysis demonstrated interactions between *CDKN1A/CDKN1B* and numerous proteins involved in G1/S phase regulation, including cyclins (e.g., CCND1, CCND2, CCND3, CCNA1, CCNA2, and CCNE1) and cyclin-dependent kinases (e.g., CDK2, CDK4, and CDK6). *CDKN1A* also showed interactions with TP53 and PCNA, key components of DNA damage response pathways **(Figure 4).**

**Figure 4:**
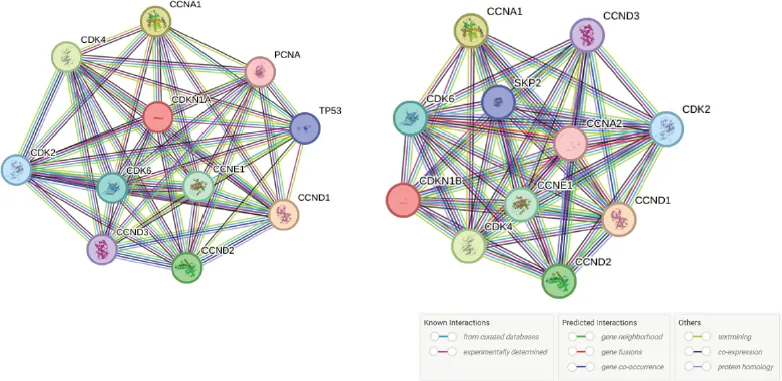
Protein-protein interaction network of *CDKN1A* (p21) and *CDKN1B* (p27) generated with the STRING database. Nodes represent proteins, and edges indicate predicted functional associations. Both inhibitors interact with cyclins (CCND1, CCNA2, CCNE1) and cyclin-dependent kinases (CDK2, CDK4, CDK6). *CDKN1A* also connects to TP53 and PCNA, consistent with their roles in G1/S checkpoint control and the DNA damage response.

## DISCUSSION

This study investigated four polymorphisms in *CDKN1A* and *CDKN1B* to clarify their contribution to genetic susceptibility for DTC. The main finding was a statistically significant association between the *CDKN1A* rs1059234 variant and DTC risk, primarily driven by a substantially higher frequency of the CT genotype among affected individuals. The observed effect size is consistent with previous reports linking rs1059234 to susceptibility in other tumor types, including gastric esophageal carcinomas (32-34), suggesting that altered post-transcriptional regulation of *CDKN1A* may broadly influence tumorigenesis. Such inflation, the so-called "winner's curse", has been described in genetic association studies, in which initial estimates of effect size may be larger than those observed in subsequent replication cohorts. Therefore, the magnitude of the association reported herein should be interpreted with caution until confirmed in larger and independent cohorts. Bioinformatic analyses supported this finding, suggesting that rs1059234 modifies miRNA binding within the 3'UTR, potentially affecting mRNA stability or translation efficiency, as previously proposed (35-37). However, without functional assays, these *in silico* predictions could not be experimentally validated in this study. Hence, functional studies, such as luciferase reporter assays or RNA-binding protein analyses, are necessary to directly determine the regulatory impact of this variant. Although functional validation is required, our findings reinforce the hypothesis that regulatory, rather than coding, variants in *CDKN1A* may contribute to carcinogenesis in hormonally regulated or highly proliferative tissues. Clinically, if confirmed in larger and independent cohorts, this polymorphism could be included in multi-marker genetic panels for identifying individuals at higher risk for thyroid cancer.

In contrast, rs1801270, a missense variant previously associated with altered cancer risk in several malignancies, including medullary thyroid carcinoma, cervical neoplasia, colorectal cancer, and retinoblastoma (^32^,^38^-40), was not associated with DTC in this cohort. *In silico* analyses suggested that the S31R substitution may affect protein stability or regulatory interactions, which is consistent with earlier predictions of its structural or functional impact. Nevertheless, these potential molecular impacts did not translate into measurable phenotypic effects, thereby underscoring the complexity genotype-phenotype relationships and suggesting that rs1801270 does not exert a major influence on DTC risk in isolation.

Regarding *CDKN1B,* neither rs2066827 nor rs34330 was associated with DTC susceptibility or tumor characteristics. These findings are consistent with the mixed results from prior studies. The rs2066827 missense variant has shown variable associations with breast, colorectal, and other cancers (41-43), albeit often inconsistently. Although *in silico* predictions suggested decreased p27 stability with the V109G substitution, this did not translate into a discernible clinical impact. Similarly, the rs34330 promoter variant, previously reported to influence mRNA expression and risk in follicular variant of papillary thyroid carcinoma (^20^), was not associated with DTC in this study. These discrepancies highlight the multifactorial nature of thyroid cancer susceptibility and suggest the effects of *CDKN1B* variants may be context-dependent and influenced by additional genetic or environmental factors.

The biological plausibility of *CDKN1A* and *CDKN1B* as susceptibility genes is strong, and both p21 and p27 play essential roles in G1/S checkpoint control, genomic stability, and DNA damage response coordination (^44^,^45^). Dysregulation of these pathways contributes to thyroid tumorigenesis, as reflected by altered expression profiles, aberrant nuclear-cytoplasmic localization, and decreased protein levels in aggressive thyroid tumors (^46^). Germline mutations in *CDKN1B* are well-established drivers of endocrine tumor predisposition in MEN4 (^47^), reinforcing the importance of p27 in endocrine homeostasis.

Despite these mechanistic insights, several limitations must be acknowledged. First, despite the sample size being sufficient to detect a strong association for rs1059234, it may have lacked adequate power to identify weaker effects or gene-environment interactions involving other variants. Second, while geno-typing call rates were generally high, incomplete data for a small subset of samples marginally reduced the effective sample size per SNP. Third, the absence of functional assays precluded experimental validation of the *in silico* predictions. An additional limitation was the lack of detailed information on environmental and lifestyle factors known to influence the risk of DTC, such as iodine intake, exposure to ionizing radiation, and family history of thyroid cancer. As these variables were not systematically available in the clinical records, they could not be included in the analyses and, as a result, residual confounding cannot be excluded.

Nevertheless, this study provides evidence that regulatory variants in *CDKN1A,* particularly rs1059234, may influence DTC susceptibility. The observed association, supported by bioinformatic evidence of altered miRNA interactions, suggests a plausible biological mechanism. Conversely, the lack of association with *CDKN1B* variants, including the structurally implicated rs2066827, underscores the need for integrative approaches combining genetic, transcriptomic, and functional data to elucidate the role of CDK inhibitor pathways in thyroid carcinogenesis.

If validated in larger, independent cohorts, the rs1059234 polymorphism could inform more precise thyroid cancer risk assessment and aid in identifying individuals who may benefit from enhanced surveillance or earlier intervention. Incorporating such genetic markers into clinical management algorithms has the potential to improve outcomes through personalized follow-up protocols.

In summary, rs1059234 in *CDKN1A* was the only variant significantly associated with DTC in this cohort, reinforcing the importance of post-transcriptional regulation of p21 in thyroid tumorigenesis. However, replication in larger, independent cohorts and functional validation are required before this polymorphism can be considered for genetic risk stratification. The *CDKN1B* polymorphisms evaluated in this study did not influence susceptibility or tumor characteristics.

## Data Availability

datasets related to this article will be available upon request to the corresponding author.

## References

[1] Maleki Z, Hassanzadeh J, Aryaie M, Ghaem H (2025). Global trend and geographic distribution of thyroid cancer from
1990 to 2021 and its prediction until 2030. Discov Oncol.

[2] Chen DW, Haymart MR (2026). Unravelling the rise in thyroid cancer incidence and addressing
overdiagnosis. Nat Rev Endocrinol.

[3] Enewold L, Zhu K, Ron E, Marrogi AJ, Stojadinovic A, Peoples GE (2009). Rising thyroid cancer incidence in the United States by
demographic and tumor characteristics, 1980-2005. Cancer Epidemiol Biomarkers Prev.

[4] Zhao HH, Pickney C, Sarode AL, Kim-Mackow A, Wilhelm SM (2023). Varying impact of patient age on the rising rate of pediatric
thyroid cancer: Analysis of NCDB database (2004-2017). Am J Surg.

[5] Kamani T, Charkhchi P, Zahedi A, Akbari MR (2022). Genetic susceptibility to hereditary non-medullary thyroid
cancer. Hered Cancer Clin Pract.

[6] Puthanmadhom Narayanan S, Najjar YG (2022). Hereditary Cancer Syndromes-A Broader Clinical Spectrum Than
Previously Understood?. JAMA Oncol.

[7] Hanahan D, Weinberg RA (2011). Hallmarks of cancer: the next generation. Cell.

[8] Abukhdeir AM, Park BH (2008). P21 and p27: roles in carcinogenesis and drug
resistance. Expert Rev Mol Med.

[9] el-Deiry WS, Tokino T, Velculescu VE, Levy DB, Parsons R, Trent JM (1993). WAF1, a potential mediator of p53 tumor
suppression. Cell.

[10] Dutto I, Tillhon M, Prosperi E (2016). Assessing Cell Cycle Independent Function of the CDK Inhibitor
p21(CDKN(1)A) in DNA Repair. Methods Mol Biol.

[11] Mishra V, Kowtal P, Rane P, Sarin R (2019). Genetic risk association of CDKN1A and RET gene SNPs with
medullary thyroid carcinoma: Results from the largest MTC cohort and
meta-analysis. Cancer Med.

[12] Taghavi N, Biramijamal F, Abbaszadegan MR, Khademi H, Sotoudeh M, Khoshbakht S (2010). P21(waf1/cip1) gene polymorphisms and possible interaction with
cigarette smoking in esophageal squamous cell carcinoma in northeastern
Iran: a preliminary study. Arch Iran Med.

[13] Nie X, Wang X, Lin Y, Yu Y, Liu W, Xie F (2016). SNP rs1059234 in CDKN1A Gene Correlates with Prognosis in
Resected Gastric Adenocarcinoma. Clin Lab.

[14] Ryan BM, Robles AI, Harris CC (2010). Genetic variation in microRNA networks: the implications for
cancer research. Nat Rev Cancer.

[15] Eltayeb MM, Ali MM, Omar SM, Mohamed NS, Adam I, Hamdan HZ (2022). Gene polymorphisms of cyclin-dependent kinase inhibitor and
matrix metalloproteinase-9 in Sudanese patients with esophageal squamous
cell carcinoma. Mol Genet Genomic Med.

[16] Kaya Z, Karan BM, Almali N (2022). CDKN1A (p21 gene) polymorphisms correlates with age in esophageal
cancer. Mol Biol Rep.

[17] Shu XO, Moore DB, Cai Q, Cheng J, Wen W, Pierce L (2005). Association of cyclin D1 genotype with breast cancer risk and
survival. Cancer Epidemiol Biomarkers Prev.

[18] Pesutic-Pisac V, Punda A, Gluncic I, Bedekovic V, Pranic-Kragic A, Kunac N (2008). Cyclin D1 and p27 expression as prognostic factor in papillary
carcinoma of thyroid: association with clinicopathological
parameters. Croat Med J.

[19] Fedele M, Palmieri D, Chiappetta G, Pasquinelli R, De Martino I, Arra C (2009). Impairment of the p27kip1 function enhances thyroid
carcinogenesis in TRK-T1 transgenic mice. Endocr Relat Cancer.

[20] Landa I, Montero-Conde C, Malanga D, De Gisi S, Pita G, Leandro-Garcia LJ (2010). Allelic variant at -79 (C>T) in CDKN1B (p27Kip1) confers an
increased risk of thyroid cancer and alters mRNA levels. Endocr Relat Cancer.

[21] Merce F, Asla Q, Illana FJ, Victoria F, Javier HL, Marta S (2025). A novel likely pathogenic germline variant in CDKN1B in a patient
with MEN4 and medullary thyroid cancer. Fam Cancer.

[22] Ng PC, Henikoff S (2003). SIFT: Predicting amino acid changes that affect protein
function. Nucleic Acids Res.

[23] Capriotti E, Fariselli P, Casadio R (2005). I-Mutant2.0: predicting stability changes upon mutation from the
protein sequence or structure. Nucleic Acids Res.

[24] Bon L, Lucchetti C, Portolan F, Pagan M (2002). MUPRO: a multipurpose robot. Int J Neurosci.

[25] Manfredi M, Savojardo C, Martelli PL, Casadio R (2022). E-SNPs&GO: embedding of protein sequence and function
improves the annotation of human pathogenic variants. Bioinformatics.

[26] Cheng C, Peters T, Dangi A, Agrawal S, Chen H, Kothapalli SR (2022). Improving PMUT Receive Sensitivity via DC Bias and Piezoelectric
Composition. Sensors (Basel).

[27] Guo L, Du Y, Chang S, Zhang K, Wang J (2014). rSNPBase: a database for curated regulatory SNPs. Nucleic Acids Res.

[28] Guo L, Wang J (2018). rSNPBase 3.0: an updated database of SNP-related regulatory
elements, element-gene pairs and SNP-based gene regulatory
networks. Nucleic Acids Res.

[29] Bhattacharya A, Ziebarth JD, Cui Y (2014). PolymiRTS Database 3.0: linking polymorphisms in microRNAs and
their target sites with human diseases and biological
pathways. Nucleic Acids Res.

[30] Rodrigues CHM, Pires DEV, Ascher DB (2021). DynaMut2: Assessing changes in stability and flexibility upon
single and multiple point missense mutations. Protein Sci.

[31] Ben Chorin A, Masrati G, Kessel A, Narunsky A, Sprinzak J, Lahav S (2020). ConSurf-DB: An accessible repository for the evolutionary
conservation patterns of the majority of PDB proteins. Protein Sci.

[32] Barbieri RB, Bufalo NE, Secolin R, Assumpcao LV, Maciel RM, Cerutti JM (2014). Polymorphisms of cell cycle control genes influence the
development of sporadic medullary thyroid carcinoma. Eur J Endocrinol.

[33] Vargas-Torres SL, Portari EA, Silva AL, Klumb EM, Rocha Guillobel HC da, Camargo MJ de (2016). Roles of CDKN1A gene polymorphisms (rs1801270 and rs1059234) in
the development of cervical neoplasia. Tumour Biol.

[34] Datkhile KD, Bhosale SJ, Durgawale PP, Jagdale NJ, More AL, Gudur RA (2023). TP53 (rs1042522, rs28934571) and TP21 (rs1801270, rs1059234)
Polymorphisms and Risk of Breast Cancer among Rural Women of Maharashtra:
Findings from a Hospital Based Case- Control Study. Asian Pac J Cancer Prev.

[35] Manousakis E, Miralles CM, Esquerda MG, Wright RHG (2023). CDKN1A/p21 in Breast Cancer: Part of the Problem, or Part of the
Solution?. Int J Mol Sci.

[36] He C, Chen H, Liu Y, Li X, Zhang C, Qin Q (2020). miR-106b-5p promotes cell proliferation and cell cycle
progression by directly targeting CDKN1A in osteosarcoma. Exp Ther Med.

[37] Petrocca F, Visone R, Onelli MR, Shah MH, Nicoloso MS, de Martino I (2008). E2F1-regulated microRNAs impair TGFbeta-dependent cell-cycle
arrest and apoptosis in gastric cancer. Cancer Cell.

[38] Cao Q, Wang Y, Song X, Yang W (2018). Association between MDM2 rs2279744, MDM2 rs937283, and p21
rs1801270 polymorphisms and retinoblastoma susceptibility. Medicine (Baltimore).

[39] Chen R, Liu S, Ye H, Li J, Du Y, Chen L (2015). Association of p53 rs1042522, MDM2 rs2279744, and p21 rs1801270
polymorphisms with retinoblastoma risk and invasion in a Chinese
population. Sci Rep.

[40] Zali N, Savabkar S, Tajali R, Chaleshi V, Nazemalhosseini Mojarad E, Vahedi M (2023). Association between CDKN1A gene rs1801270 polymorphisms and
susceptibility to colorectal cancser in an Iranian
population. Nucleosides Nucleotides Nucleic Acids.

[41] Lu Y, Gao K, Zhang M, Zhou A, Zhou X, Guan Z (2015). Genetic Association Between CDKN1B rs2066827 Polymorphism and
Susceptibility to Cancer. Medicine (Baltimore).

[42] Xiang H, Li H, Ge W, Wu W, Gao M, Wang W (2013). Association of CDKN1B gene polymorphisms with susceptibility to
breast cancer: a meta-analysis. Mol Biol Rep.

[43] Spurdle AB, Deans AJ, Duffy D, Goldgar DE, Chen X, Beesley J (2009). No evidence that CDKN1B (p27) polymorphisms modify breast cancer
risk in BRCA1 and BRCA2 mutation carriers. Breast Cancer Res Treat.

[44] Malumbres M, Barbacid M (2009). Cell cycle, CDKs and cancer: a changing paradigm. Nat Rev Cancer.

[45] Anand SK, Sharma A, Singh N, Kakkar P (2020). Entrenching role of cell cycle checkpoints and autophagy for
maintenance of genomic integrity. DNA Repair (Amst).

[46] Erickson LA, Yousef OM, Jin L, Lohse CM, Pankratz VS, Lloyd RV (2000). p27kip1 expression distinguishes papillary hyperplasia in Graves'
disease from papillary thyroid carcinoma. Mod Pathol.

[47] Mazarico-Altisent I, Capel I, Baena N, Bella-Cueto MR, Barcons S, Guirao X (2023). Novel germline variants of CDKN1B and CDKN2C identified during
screening for familial primary hyperparathyroidism. J Endocrinol Invest.

